# Pre-Diabetes Increases Tuberculosis Disease Severity, While High Body Fat Without Impaired Glucose Tolerance Is Protective

**DOI:** 10.3389/fcimb.2021.691823

**Published:** 2021-07-06

**Authors:** Roma Sinha, Minh Dao Ngo, Stacey Bartlett, Helle Bielefeldt-Ohmann, Sahar Keshvari, Sumaira Z. Hasnain, Meg L. Donovan, Jessica C. Kling, Antje Blumenthal, Chen Chen, Kirsty R. Short, Katharina Ronacher

**Affiliations:** ^1^ Translational Research Institute, Mater Research Institute, The University of Queensland, Brisbane, QLD, Australia; ^2^ School of Chemistry and Molecular Biosciences, The University of Queensland, Brisbane, QLD, Australia; ^3^ Australian Infectious Diseases Research Centre – The University of Queensland, Brisbane, QLD, Australia; ^4^ The University of Queensland Diamantina Institute, Brisbane, QLD, Australia; ^5^ School of Biomedical Sciences, The University of Queensland, Brisbane, QLD, Australia

**Keywords:** impaired glucose tolerance, high fat diet, disease severity, pre-diabetes, diabetes, *Mycobacterium tuberculosis*, tuberculosis, interferon responses

## Abstract

Type 2 diabetes (T2D) is a well-known risk factor for tuberculosis (TB), but little is known about pre-diabetes and the relative contribution of impaired glucose tolerance *vs.* obesity towards susceptibility to TB. Here, we developed a preclinical model of pre-diabetes and TB. Mice fed a high fat diet (HFD) for 12 weeks presented with impaired glucose tolerance and hyperinsulinemia compared to mice fed normal chow diet (NCD). Infection with *M. tuberculosis* (Mtb) H_37_R_v_ after the onset of dysglycemia was associated with significantly increased lung pathology, lower concentrations of TNF-α, IFN-γ, IFN-β and IL-10 and a trend towards higher bacterial burden at 3 weeks post infection. To determine whether the increased susceptibility of pre-diabetic mice to TB is reversible and is associated with dysglycemia or increased body fat mass, we performed a diet reversal experiment. Pre-diabetic mice were fed a NCD for 10 additional weeks (HFD/NCD) at which point glucose tolerance was restored, but body fat mass remained higher compared to control mice that consumed NCD throughout the entire experiment (NCD/NCD). Upon Mtb infection HFD/NCD mice had significantly lower bacterial burden compared to NCD/NCD mice and this was accompanied by restored IFN-γ responses. Our findings demonstrate that pre-diabetes increases susceptibility to TB, but a high body mass index without dysglycemia is protective. This murine model offers the opportunity to further study the underlying immunological, metabolic and endocrine mechanisms of this association.

## Introduction

Tuberculosis (TB) remains one of the top 10 causes of death worldwide killing more than 1.4 million people in 2019 (WHO, 2020). Type 2 diabetes (T2D) increases the risk of developing TB as well as the risk of adverse TB treatment outcomes (Critchley et al., 2017). People with TB and T2D co-morbidity have a 88% higher risk of death during treatment, a 64% higher risk of relapse and are twice as likely to develop drug-resistant TB (Huangfu et al., 2019). Paradoxically, obesity in absence of dysglycemia protects against TB (Lonnroth et al., 2010; Aibana et al., 2016; Lin et al., 2018) and individuals with high BMI are less likely to die during TB treatment (Yen et al., 2016).

Increased susceptibility of T2D patients to TB has been attributed to poor glycemic control (Critchley et al., 2018). However, immune dysfunction and altered immunity to TB has also been demonstrated in individuals with pre-diabetes (Kumar et al., 2014; Eckold et al., 2020). Strikingly, blood transcriptomic profiles of TB patients with pre-diabetes are more similar to TB patients with T2D than those without any form of dysglycemia (Eckold et al., 2020). Whether pre-diabetes increases susceptibility to TB and TB disease severity remains unknown and it is also not clear which immunological mechanisms underlie obesity associated resistance vs. diabetes associated susceptibility to TB.

Several different animal models of TB and type 1 or type 2 diabetes have been established to study the underlying immunological mechanisms of diabetes-induced increased susceptibility to TB (Yamashiro et al., 2005; Martens et al., 2007; Sugawara and Mizuno, 2008; Vallerskog et al., 2010; Podell et al., 2014; Martinez et al., 2016; Tripathi et al., 2019; Alim et al., 2020). Such animal models are particularly useful to study immune responses at the site of infection, the lung, which is difficult to achieve in patients. Despite differences in species and methods used for inducing diabetes, these studies demonstrate a clear association between diabetes and increased susceptibility to TB. Diabetic animals have higher bacterial loads, more severe tissue pathology and reduced survival. Therefore, these animal models mimic clinical observations from individuals with TB and diabetes co-morbidity. Whether pre-diabetes impacts susceptibility to TB has not been extensively investigated in animal models with only one study from guinea pigs (Podell et al., 2014). Most importantly, no published data exist relating to the relative contribution of dysglycemia and obesity in susceptibility or resistance to TB. Given the high global prevalence rates of pre-diabetes in TB household contacts from both low and high TB burden countries - with 23% and 25% in South Africa and South Texas, respectively (Restrepo et al., 2018) - it is imperative to expand TB and diabetes association studies to include obesity with and without dysglycemia.

Here, we developed a murine model of high fat-diet (HFD)-induced pre-diabetes and a diet reversal model to dissect the relative contribution of dysglycemia vs. obesity to susceptibility TB. Pre-diabetic mice had more severe TB and dysregulated cytokine production both at the site of infection and in the periphery, while obese animals with restored glucose tolerance were more resistant to TB.

## Materials and Methods

### Ethics Statement

All experiments were carried out in accordance with protocols approved by the Health Sciences Animal Ethics Committee of The University of Queensland (MRI-UQ/413/17) and performed in accordance with the Australian Code of Practice for the Care and Use of Animals for Scientific Purposes.

### Murine Pre-Diabetes and Diet-Reversal Models

Six-week-old male C57BL/6 mice were housed in a conventional pathogen free environment, on a 12-hour light/dark cycle at 22°C and fed *ad libitum*. Male mice were chosen for this study as they are more susceptible to developing HFD-induced hyperglycemia (Heydemann, 2016). Animals were either fed a lard based-HFD for 12 weeks (HFD), which contained 43% available energy as fat (total fat: 23.50%, SF04-001, Specialty Feeds, Western Australia) or normal chow diet (NCD) with 12% available energy from fat for the same period (total fat: 4.60%, Standard rodent diet, Specialty Feeds, Western Australia). For the diet reversal experiment 12-week HFD fed animals were fed NCD diet for a further 10 weeks (HFD/NCD) while the control group continued on a NCD for the same period of time (NCD/NCD). Body weights of all mice were recorded weekly throughout the experiment. The respective diets continued until conclusion of the experiment. Mice were infected with Mtb H_37_R_v_ at week 12 or 22 as described below.

### Oral Glucose Tolerance Test, HbA1c and Insulin Measurement

At 12 or 22 weeks on the respective diets, oral glucose tolerance tests (OGTT), fasting insulin measurements and body composition analyses were performed. Mice were fasted for 5h with access to drinking water followed by oral gavage with a fixed dose of 50 mg glucose per mouse which has been proved sufficient to show glucose intolerance in mice irrespective of body weight (Andrikopoulos et al., 2008). Blood was collected from tail veins and glucose concentrations were determined using a glucometer (Sensocard Plus, Elektronika Kft., Budapest, Hungary) before (0 min) and at 15, 30, 60, and 120 min after gavage. HbA1c was measured from total blood using the DCA Vantage analyzer (Siemens Healthcare Diagnostics Inc., Germany). Fasting insulin levels were quantified in serum using Ultra-Sensitive Mouse Insulin ELISA Kit (Crystal Chem, IL, USA) as per manufacturer’s instruction.

### Body Composition Measurements and Physiological Monitoring

Whole body composition (fat and lean mass) was measured using a Bruker Minispec LF50H NMR instrument 7.5 MHz (Bruker Corporation, MA, USA) (Tinsley et al., 2004). A subset of five mice per group from the diet change experiment (at week 12 and week 22) were housed in single caging for 1 week for acclimatization followed by 3 days of metabolic profiling using the PhenoMaster System (TSE systems GmbH, Bad Homburg, Germany). Energy expenditures including CO_2_ production (VCO_2_) and O_2_ consumption (VO_2_) were monitored for 72h and respiratory exchange ratio (RER) was calculated VCO_2_/VO_2_. The mice were free to consume food and intake was measured. The resting energy expenditure (REE) was calculated using Weir equation (Weir, 1949).

### Mtb Infection, Determination of Bacterial Burden and Immunopathology

Mtb H_37_R_v_ was grown on Middlebrook 7H9 medium containing 0.05% Tween-80 and supplemented with 10% Middlebrook Oleic Albumin Dextrose Catalase Growth Supplement (BD Biosciences, USA)/0.2% glycerol to mid-log phase (OD_600_ 0.4 to 0.6). On the day of infection, a single cell suspension was prepared (O.D. of 0.1, equivalent to 50 million cells/ml) and placed in a nebulizer of an inhalation exposure system (Glas-Col, LLC, IN, USA) for aerosol infection of mice. Approximately 100-150 colony forming units (CFU) were deposited in the lung. Lungs, livers, spleens and blood were collected for determination of bacterial counts, pathology, RNA extractions and cytokine analysis as described below. For bacterial load determination tissues were homogenized, serially diluted in PBS and plated on 7H10 agar plates supplemented with 10% OADC/0.5% glycerol and incubated at 37°C. Bacterial colonies were counted after 2-3 weeks. Formalin-fixed lung lobe sections were stained with hematoxylin and eosin (H&E). A qualified pathologist analyzed images of H&E-stained sections from lungs, without prior knowledge of the groupings, as previously described (Flores-Valdez et al., 2018). Briefly, the number of lesions apparent in a section was counted and the percentage of involved parenchyma estimated and assigned an extent score as follows: <10% = 1; 10-20% = 2; 21-30% = 3; 31-50% = 4; >50% = 5. The following features were assessed individually: peribronchiolitis, perivascular leukocyte infiltration, perivasculitis, alveolitis, “granuloma” formation (i.e., granulomatous inflammation), and necrosis on a scale of 0–5 [0 = within normal limits (no change); 1 = minimal changes; 2 = mild changes; 3 = moderate changes; 4 = marked changes; 5 = very severe changes]. In addition, the proportion of macrophages with foamy cytoplasm within the lesions was assessed on a scale of 0 to 5.

### RNA Extraction and qRT-PCR

RNA was isolated from lung and blood using Isolate II RNA mini kit protocol (Bioline Reagents Ltd., London, UK) with slight modification. Briefly, blood cell pellet and lung lobes were homogenized in Trizol and vigorously mixed with chloroform (2.5:1) and centrifuged at 12,000 *x* g for 15 min at 4°C. The RNA in the aqueous phase was precipitated by mixing in cold 70% ethanol (1:2.5) followed by column-based RNA isolation using kit protocol including DNase treatment to remove genomic DNA contamination. Complementary DNA was synthesized using 2 μg of RNA and the Tetro cDNA synthesis kit (Bioline Reagents Ltd., London, UK) according to manufacturer’s instructions. Gene expression analysis was performed by quantitative real time PCR (qRT-PCR) with SensiFAST™ SYBR^®^ Lo-ROX Kit (Bioline Reagents Ltd., London, UK) run on the QuantStudio™ 7 Flex Real-Time PCR System (Applied Biosystems). All gene expression levels were normalized to *Hprt1* internal controls in each sample, and the fold changes were calculated using the 2^–ΔΔCT^ method. The list of primers used is given in **Table S1**.

### ELISA

Lung homogenate supernatants were collected by centrifugation at 2000 *x* g at 4°C and stored at -80°C with protease inhibitor cocktail (Sigma). Quantification of TNF-α, IL-1β, IFN-β, CCL2, IFN-γ, and IL-10 were performed by ELISA according to the manufacturer’s instructions (R&D Systems).

### Statistical Analysis

Data analyses were performed using GRAPHPAD PRISM Version 8 (GraphPad Software, Inc., La Jolla, CA). The results are expressed as the mean ± SEM. Comparisons between two groups were performed using non-parametric unpaired Mann-Whitney *U*-test. The relationship between two variables was ranked using Spearman’s rank correlation coefficient. Statistically significant differences between two groups are indicated in the figures as follows *, p < 0.05; **, p < 0.01; ***, p < 0.001; ****, p < 0.0001.

## Results

### Pre-Diabetes Increases TB Severity

**Figure 1:**
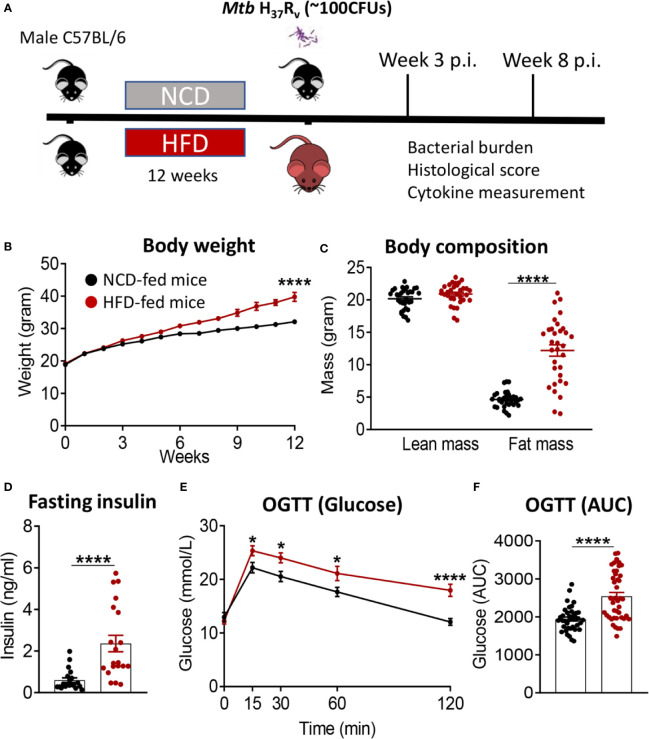
Murine model of pre-diabetes. **(A)** Experimental design. **(B)** Body weight of mice fed NCD (black) or HFD (red) (n=30mice/group); **(C)** Body fat and lean mass at 12 weeks; **(D)** fasting insulin at 12 weeks (n=20 mice/group); **(E)** Blood glucose concentrations at baseline, 15, 30, 60, 120 minutes after oral glucose administration; **(F)** OGTT Area under curve (AUC) (n=30 mice/group). Data are means ± SEM. Data analysis was performed by Mann-Whitney *U* test. *p < 0.05 and ****p < 0.0001.

We developed a murine model of pre-diabetes and Mtb infection. C57BL/6 mice were fed HFD or NCD for a period of 12 weeks (**Figure 1A**). HFD-fed mice had significantly higher body weight (**Figure 1B**) and body fat mass but similar lean mass (**Figure 1C**) compared to NCD-fed mice. HFD-fed mice developed hyperinsulinemia indicative of insulin resistance (**Figure 1D**). Blood glucose concentrations after glucose challenge were higher at 15, 30, 60 and 120 min in HFD-fed mice (**Figure 1E**) with significantly higher area under the curve (AUC) in OGTTs (**Figure 1F**), while fasting blood glucose and glycated hemoglobin (HbA1c) were similar between NCD and HFD-fed mice (**Figure S1**). This phenotype of obesity combined with dysglycemia therefore mimics human pre-diabetes, which is characterized by insulin resistance and impaired glucose tolerance, but HbA1c levels below those of diabetes patients.

**Figure 2:**
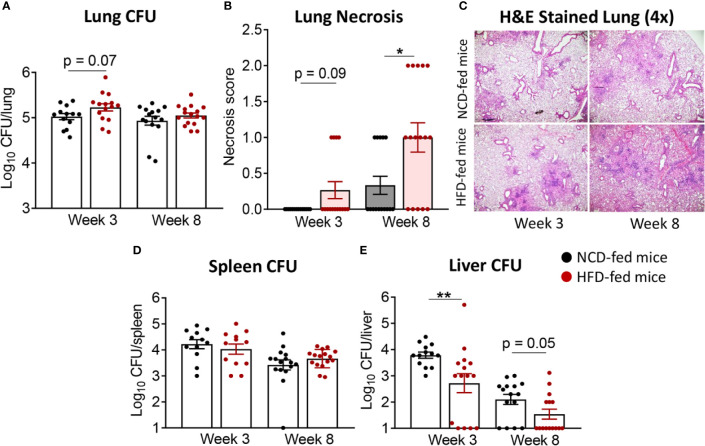
Pre-diabetes increases lung pathology in Mtb-infected mice. **(A)** Lung Mtb burden in NCD- (black) and HFD-fed mice (red) at 3- and 8-weeks p.i.; **(B)** Lung Necrosis scores; **(C)** Representative lung histological images at 4x magnification; **(D)** Mtb burden in spleen and **(E)** in liver. Data are means ± SEM (n=13-16 mice/group) analyzed cumulatively in two independent experiments. Data analysis was performed by Mann-Whitney *U* test. ns = not significant *p < 0.05, and **p < 0.01.

We subsequently infected the mice with live Mtb H_37_R_v_. At 3 weeks post infection (p.i.), mice with pre-diabetes had higher lung Mtb burden, although this did not reach significance (p = 0.07, **Figure 2A**). Lung necrosis appeared at 3 weeks p.i. in HFD-fed mice while necrosis was not detectable at this early timepoint in any NCD-fed mice. Necrosis scores were significantly higher in pre-diabetic mice by 8 weeks p.i. (**Figures 2B, C**) demonstrating increased lung immunopathology associated with pre-diabetes. Bacterial loads in spleens were comparable between pre-diabetic and control mice (**Figure 2D**). Interestingly, the Mtb burden in the fatty livers of HFD-fed mice was significantly lower than in NCD-fed mice at 3 weeks p.i, and this trend continued at 8 weeks p.i. (**Figure 2E**). Our data demonstrate that obesity with impaired glucose tolerance below the threshold of diabetes, i.e., pre-diabetes, increases susceptibility to pulmonary TB in a murine model.

### Pre-Diabetes Alters the Immune Response to Mtb in the Lung

**Figure 3:**
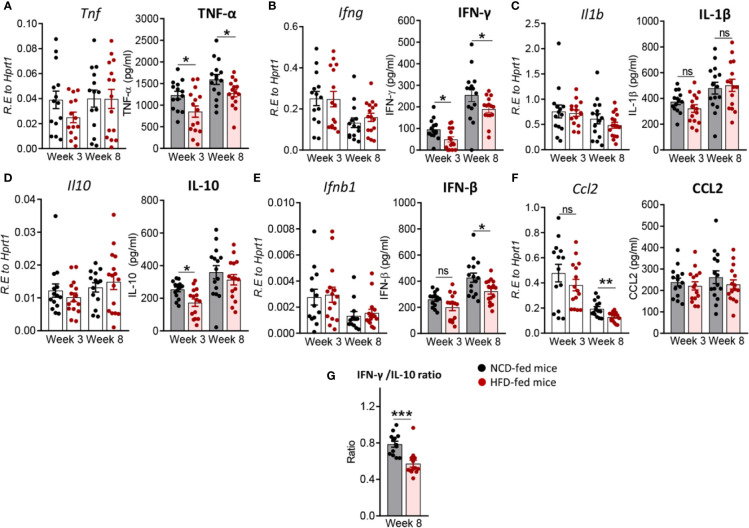
Pre-diabetes alters cytokine production in Mtb-infected lungs. Cytokine mRNA and protein levels were determined in lung homogenates by qRT-PCR and ELISA. Lung mRNA expression and protein concentrations of **(A)** *Tnf*, TNF-α; **(B)** *Ifng*, IFN-γ; **(C)** *Il1b*, IL-1β; **(D)** *Il10*, IL-10; **(E)** *Ifnb1*, IFN-β and **(F)** *Ccl2*, CCL2 from NCD- and HFD fed-mice at 3-and-8 weeks p.i. **(G)** IFN-γ/IL-10 ratio was determined for each mouse at week 8. Data are means ± SEM of n=13-15 mice/group analyzed cumulatively across two independent experiments. Data analysis was performed by Mann-Whitney *U* test. ns = not significant, *p < 0.05, and ***p < 0.001.

Next, we investigated whether immune responses to Mtb at the site of infection are modified by pre-diabetes. We determined relative cytokine and chemokine mRNA expression and protein concentrations in lung homogenates from obese mice with impaired glucose tolerance and healthy control animals at 3 and 8 weeks p.i. The mRNA expression of *Tnf, Ifng, Il1b, Ifnb1* and *Il10* was similar between animals (**Figures 3A–E**), however, mRNA expression of the chemokine *Ccl2* was significantly lower in pre-diabetic mice compared to controls at 8 weeks p.i. (**Figure 3F**). At the protein level, TNF-α, IFN-γ and IL-10 were significantly lower at 3 weeks p.i. in pre-diabetic mice compared to control animals (**Figures 3A, B, D**) and concentrations of TNF-α and IFN-γ remained lower also at 8 weeks p.i. IFN-β was significantly lower in HFD-fed mice compared to NCD-fed mice at 8 weeks p.i. (**Figure 3E**), but IL-1β and CCL2 were not different between the groups (**Figures 3C, F**). TGF-β, IL-6 and IL-12 mRNA expression and protein concentrations were not impacted by HFD (data not shown). In uninfected animals, no significant differences in cytokine transcript levels between NCD- and HFD-fed mice were observed at both 3-and-8 weeks p.i. (**Figure S2**). A low IFN-γ/IL-10 ratio is a biomarker for increased TB disease severity in humans (Jamil et al., 2007) and this ratio was significantly lower in pre-diabetic mice compared to control animals at 8 weeks p.i. (**Figure 3G**). As expected, higher lung concentrations of TNF-α, IFN-γ, IL-1β, and CCL2 were associated with lower Mtb burden in NCD-fed mice, while this relationship was surprisingly inversed in pre-diabetic animals (**Figure S3**).

### Pre-Diabetes Alters the Immune Response to Mtb in the Periphery

**Figure 4:**
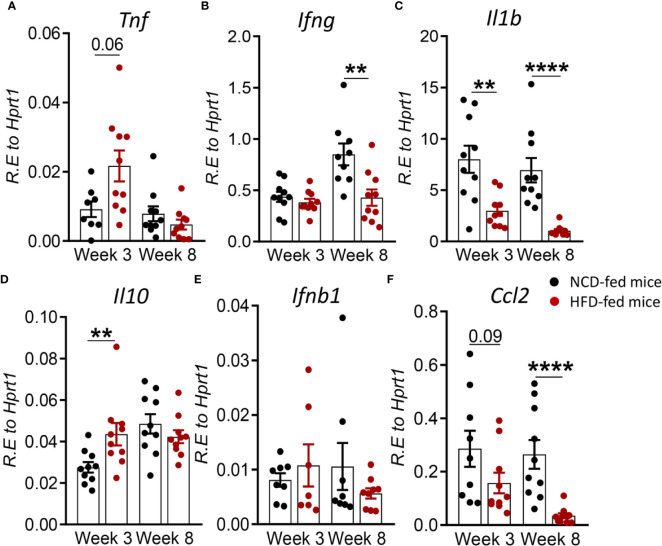
Pre-diabetes alters cytokine responses to Mtb in the periphery. Cytokine mRNA expression was determined by qRT*-*PCR in blood from NCD- and HFD-fed mice at 3- and 8-weeks p.i. **(A)** *Tnf*, **(B)** *Ifng*, **(C)** *Il1b*, **(D)** *Il10*, ***(*E*)*** *Ifnb1*, and **(F)** *Ccl2*. Data are means ± SEM (n=7-10 mice/group analyzed in one independent experiment). Data analysis was performed by Mann-Whitney *U* test. ns = not significant **p < 0.01, and ****p < 0.0001.

To determine whether changes in the immune response to Mtb infection in pre-diabetes are limited to the site of infection or also occur in the periphery, we assessed cytokine and chemokine expression in blood from HFD- and NCD-fed mice. We found that at 3 weeks p.i. *Tnf* was higher in HFD-fed mice, but this did not reach statistical significance (p = 0.06, **Figure 4A**). *Il1b* was significantly lower (**Figure 4C**), while *Il10* was significantly higher in pre-diabetic mice compared to control animals (**Figure 4D**). At 8 weeks p.i. *Ifng, Il1b* and *Ccl2* were significantly lower in HFD-fed mice (**Figures 4B, C, F**) and we did not observe any differences in *Ifnb1* expression in HFD- *vs.* NCD-fed animals (**Figure 4E**). At baseline, uninfected HFD-fed mice had reduced blood transcript levels of *Ifng*, *Il1b*, *Ccl2* but increased *Il10* compared to NCD-fed mice at both 3-and-8 weeks p.i. (**Figure S4**). These data demonstrate that pre-diabetes-induced changes in the immune response to Mtb are not confined to the lung and occur also in the periphery.

### Restored Glucose Tolerance With Elevated Body Fat Mass Confers Mild Resistance to TB

**Figure 5:**
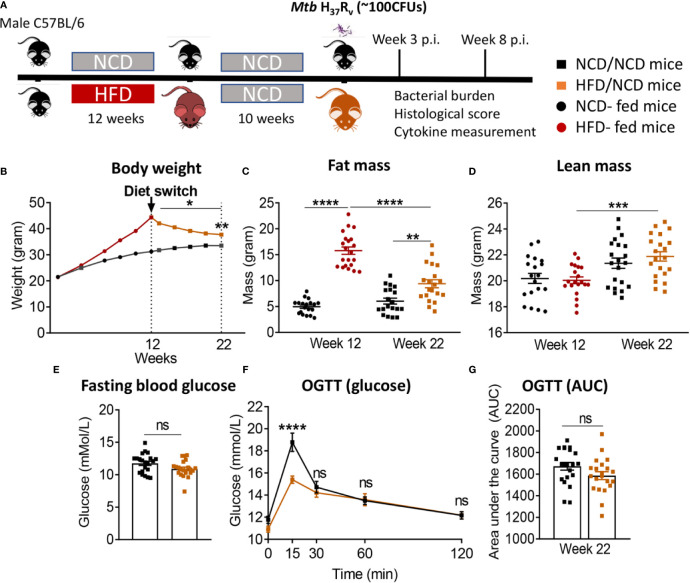
Diet reversal restores glucose tolerance while maintaining higher fat mass. **(A)** Schematic showing experimental plan for diet reversal-TB model development. **(B)** Body weight of mice were monitored up to 22 weeks of diet; **(C)** Fat mass and **(D)** lean mass were measured. OGTT was performed on NCD/NCD and HFD/NCD mice; **(E)** Fasting blood glucose **(F)** Blood glucose concentrations at baseline, 15, 30, 60, 120 minutes after oral glucose administration and **(G)** Area under curve (AUC). Data represents mean ± SEM, (n=19-20 mice/group). Data analysis was performed by Mann-Whitney *U* test. ns=not significant, *p < 0.05, **p < 0.01, ***p < 0.001 and ****p < 0.0001.

In order to assess whether a change in diet can restore glucose tolerance and reverse susceptibility to TB, we established a diet reversal model. Mice fed HFD for 12 weeks (as described for the experiment above) were subsequently fed NCD for an additional 10 weeks, here referred to as HFD/NCD mice. Control mice (NCD/NCD) were fed NCD for the entire 22 weeks (**Figure 5A**). Diet reversal resulted in significant loss of total body weight (**Figure 5B**) and fat mass (**Figure 5C**) in HFD/NCD animals, while the lean mass increased over time (**Figure 5D**). However, HFD/NCD mice maintained significantly higher body weight (**Figure 5B**) and higher body fat mass (**Figure 5C**) compared to NCD/NCD mice. Diet reversal restored the average RER and decreased the REE across 24h of light/dark phases observed in NCD-fed mice (**Figure S5**). Most importantly, diet reversal resulted in complete restoration of glucose tolerance between HFD/NCD and NCD/NCD animals (**Figures 5E–G**). We next assessed the impact of this metabolic phenotype of restored glucose tolerance but elevated body fat mass on susceptibility to TB.

**Figure 6:**
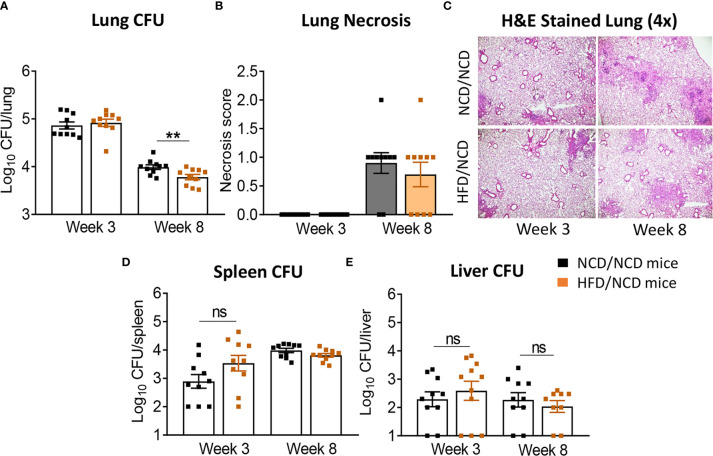
Restoration of glucose tolerance with elevated fat mass confers mild resistance to TB. **(A)** Lung Mtb burden in NCD/NCD- (black) and HFD/NCD mice (orange) at 3- and 8-weeks p.i.; **(B)** Lung Necrosis scores; **(C)** Representative lung histological images; **(D)** Mtb burden in spleen and **(E)** in liver. Data are means ± SEM (n=10 mice/group analyzed in one independent experiment). Data analysis was performed by Mann-Whitney *U* test. ns = not significant. **p < 0.01.

**Figure 7:**
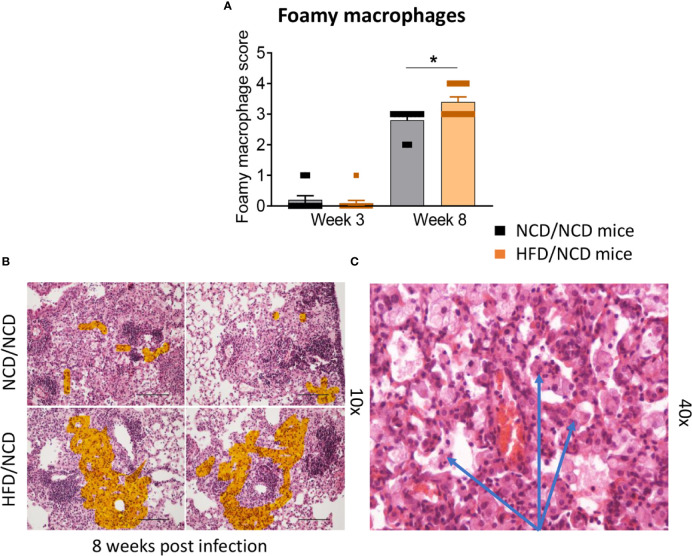
Foamy macrophages were upregulated in the lung of HFD/NCD mice. **(A)** Histological scoring of foamy macrophages in lung sections of NCD/NCD mice and HFD/NCD mice at 3-and-8 weeks p.i. **(B)** Representative histological images with highlighted area (orange) of foamy macrophages; **(C)** Snapshot of foamy macrophages on H&E lung sections (arrows). Data represents mean ± SEM (n=10 mice/group analyzed in one independent experiment). Data analysis was performed by Mann-Whitney *U* test. *p < 0.05.

HFD/NCD and NCD/NCD mice were infected with Mtb H_37_R_v_ and Mtb burden was determined at 3 and 8 weeks p.i. Interestingly, the metabolic phenotype induced by the diet reversal, i.e., restored glucose tolerance with increased body fat mass compared to controls, conferred a mild but statistically significant resistance to TB. HFD/NCD mice had significantly lower lung Mtb burden at 8 weeks p.i. compared to control animals that only consumed NCD throughout the entire experiment (**Figure 6A**), while Mtb burden was similar at 3 weeks p.i. No significant differences were observed in lung necrosis scores or spleen and liver CFU (**Figures 6B–E**). Coinciding with significantly reduced Mtb burden in the lung at 8 weeks p.i., we observed more foamy macrophages in lung sections from normoglycemic, obese mice compared to control animals (**Figure 7**) which was not evident in dysglycemic obese mice (**Figure S7**).

### Restoration of Glucose Tolerance Improves Immune Responses to Mtb in the Lung

**Figure 8:**
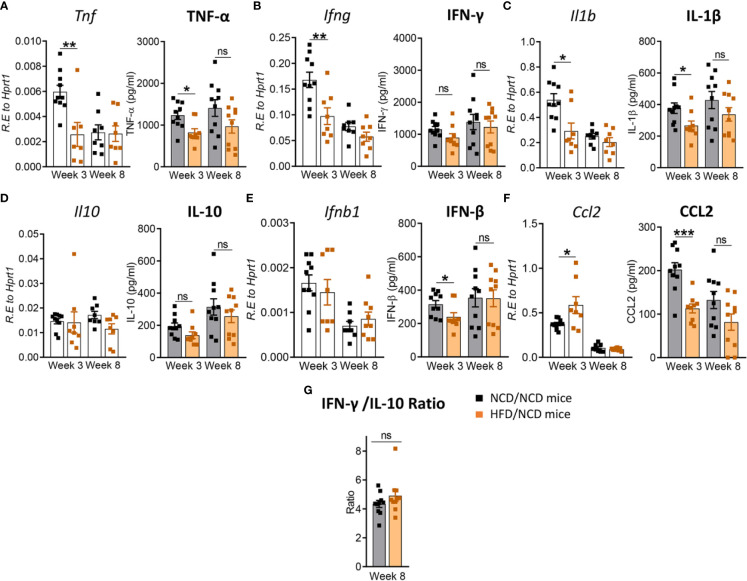
Restoration of glucose tolerance improves lung cytokine profiles. Cytokine mRNA and protein levels were determined in lung homogenates by qPCR and ELISA. Lung mRNA expression and protein concentrations of **(A)** *Tnf*, TNF-α; **(B)** *Ifng*, IFN-γ; **(C)** *Il1b*, IL-1β; **(D)** *Il10*, IL-10; **(E)** *Ifnb1*, IFN-β; and **(F)** *Ccl2*, CCL2 from NCD- and HFD fed-mice at 3- and 8-weeks p.i. **(G)** IFN-γ/IL-10 ratio was determined for each mouse at week 8. Data are means ± SEM (n=8-10 mice/group analyzed in one independent experiment). Data analysis was performed by Mann-Whitney *U* test. ns = not significant *p < 0.05, ** p<0.01 and ***p < 0.001.

We next assessed whether the change in diet and restoration of glucose tolerance while maintaining high body fat mass improves host protective immune responses to Mtb in the lung. While lung TNF-α and IFN-γ concentrations were significantly lower in pre-diabetic mice compared to control mice at both 3 and 8 weeks p.i. (**Figures 3A, B**), concentrations of these cytokines were similar in mice with restored glucose tolerance (HFD/NCD) and their respective controls (NCD/NCD) at 8 weeks p.i., although TNF-α concentrations were still lower at 3 weeks p.i. (**Figures 8A, B**). At the mRNA level *Tnf*, *Ifng* and *Il1b* were lower in HFD/NCD *vs* NCD/NCD animals (**Figures 8A–C**). This demonstrates that production of these key cytokines for protective immune responses against Mtb was restored at the protein level by the change in diet. Similarly, IL-10 production was significantly lower in HFD-fed mice compared to NCD-fed mice at 3 weeks p.i. (**Figure 3D**), but after diet reversal IL-10 concentrations were comparable between HFD/NCD and NCD/NCD animals (**Figure 8D**). IL-1β and CCL2 concentrations, which were similar in pre-diabetic and control mice (**Figures 3C, F**), were significantly lower in HFD/NCD mice compared to NCD/NCD animals at 3 weeks p.i. (**Figures 8C, F**). While IFN-β concentrations were lower in pre-diabetic mice at 8 weeks p.i. (**Figure 3E**), they were lower in HFD/NCD fed mice compared to control animals at 3 weeks p.i. (**Figure 8E**). Correlation analysis of cytokine concentrations and lung Mtb burden are shown in **Figure S6**. Most importantly, the IFN-γ/IL-10 ratio, which was significantly lower in pre-diabetic *vs.* control mice (**Figure 3G**), was now similar in animals with restored glucose tolerance and their controls (**Figure 8G**).

These data demonstrate that diet reversal significantly improves this biomarker of TB disease severity.

### Restoration of Glucose Tolerance Improves Immune Responses to Mtb in the Periphery

**Figure 9:**
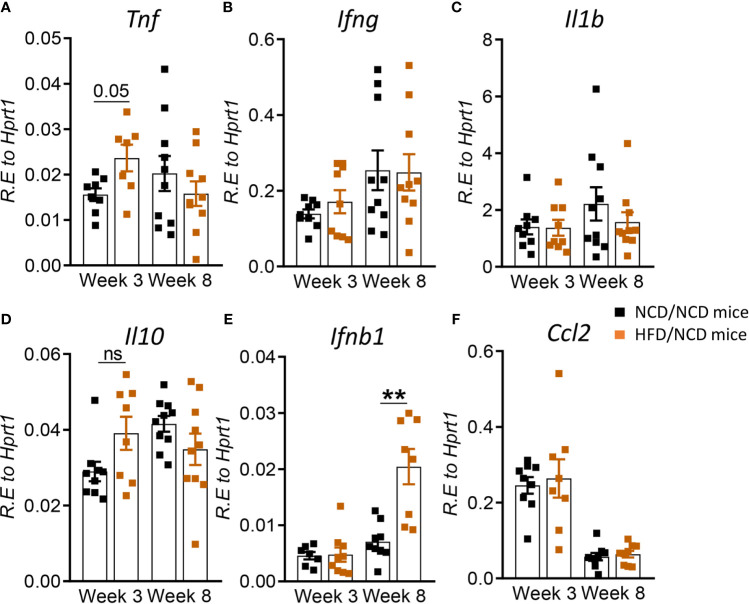
Restoration of glucose tolerance improves blood cytokine profiles. Cytokine mRNA expression was determined by qRT-PCR in blood from NCD- and HFD-fed mice at 3- and 8-weeks p.i. **(A)** *Tnf*, **(B)** *Ifng*, **(C)** *Il1b*, **(D)** *Il10*, **(E)** *Ifnb1*, and **(F)** *Ccl2*. Data are means ± SEM (n=8-10 mice/group analyzed in one independent experiment). Data analysis was performed by Mann-Whitney *U* test. ns = not significant **p < 0.01.

After diet reversal we found that cytokine mRNA expression in whole blood was restored or in the case of *tnfa* unchanged (**Figure 9A**) to those in control animals. For instance, mRNA expression of *Ifng, Il1b* and *Ccl2* which were lower in pre-diabetic mice *vs.* controls at week 8 (**Figures 4B, C, F**), but were similar in HFD/NCD vs. NCD/NCD animals (**Figures 9B, C, F**). *Il10* expression was higher in pre-diabetic animals at 3 weeks p.i. (**Figure 4D**) and was not significantly different in HFD/NCD *vs.* NCD/NCD animals (**Figure 9D**). Interestingly, blood *Ifnb* mRNA expression was significantly higher in obese mice with restored glucose tolerance compared to controls at 8 weeks p.i. (**Figure 9E**). Together these data demonstrate that HFD significantly impacts immune responses to Mtb at the site of infection, the lung, as well as the periphery and thus can contribute to TB disease severity.

## Discussion

Increased susceptibility of T2D patients to TB is well established, however, whether pre-diabetes also predisposes to more severe manifestations of pulmonary TB remains elusive as large population-based studies on the association of pre-diabetes and TB have not been performed to date. Blood transcriptomic signatures from TB patients with pre-diabetes are more similar to those from TB patients with T2D compared to TB patients without any form of dysglycemia (Eckold et al., 2020). This suggests that impaired immune responses to Mtb occur already during the early stages of dysglycemia in pre-diabetes. Given the high prevalence rates of pre-diabetes in TB endemic countries, with 27% of TB contacts in India (Shivakumar et al., 2018) and 25% in South Africa (Restrepo et al., 2018) having impaired glucose tolerance, it is imperative to investigate any associations between pre-diabetes and susceptibility to TB. To address this current knowledge gap, we developed a pre-diabetes model of Mtb infection and demonstrated more severe TB disease and altered immune responses to Mtb in the lung and blood of mice with impaired glucose tolerance.

Several different animal models of diabetes and TB exist and generally show, similarly to our pre-diabetes murine model, more severe disease and impaired immune responses in hyperglycemic hosts upon Mtb infection (Yamashiro et al., 2005; Martens et al., 2007; Sugawara and Mizuno, 2008; Vallerskog et al., 2010; Podell et al., 2014; Martinez et al., 2016; Tripathi et al., 2019; Alim et al., 2020). Many of these models use Streptozotocin (STZ) to induce hyperglycemia, which does not accurately reflect the chronic inflammation and vascular complications associated with human T2D. Nevertheless, these models provide valuable insight into hyperglycemia associated immune impairment. STZ-induced chronic hyperglycemia resulted in increased Mtb lung burden, more inflammation and lower IFN-γ production in the lung (Martens et al., 2007). Similarly, we found lower IFN-γ and TNF-α production in the lungs of pre-diabetic mice combined with more severe immunopathology and significantly lower IFN-γ/IL-10 ratios, a biomarker for TB disease severity (Jamil et al., 2007). The increased susceptibility of STZ-treated mice was attributed to a delayed innate immune response due to impaired recognition of Mtb by alveolar macrophages from hyperglycemic animals, which subsequently results in delayed adaptive immune responses (Martinez et al., 2016). It is likely that pre-diabetic mice also have a delayed adaptive immune response given the lower IFN-production, however, whether this is due to impaired recognition of Mtb by pre-diabetic alveolar macrophages remains to be elucidated in future studies. Vallerskog *et al.* reported lower CCL2 expression in the lungs of STZ-induced hyperglycemic mice (Vallerskog et al., 2010). While pre-diabetic animals in our model showed lower *Ccl2* mRNA at 8- weeks p.i., protein concentrations of CCL2 were not significantly different. Eckhold *et al.* found reduced type I IFN responses in blood transcriptomic signatures from TB patients with pre-diabetes (Eckold et al., 2020). We did not observe reduced *Ifnb1* mRNA expression in blood, however, IFN-β concentrations were significantly reduced in lungs of pre-diabetic mice at 8 weeks p.i. A HFD-based model of T2D and TB recently demonstrated moderately higher Mtb burden in the early stages of infection at 2 weeks, but not during late infection, and reduced IFN-γ production in HFD-fed diabetic mice (Alim et al., 2020), which is consistent with our data in this pre-diabetes model. An interesting observation in our murine pre-diabetes model was the significantly reduced Mtb burden in the liver of HFD-fed animals. This finding is in line with human studies showing that diabetes does not increase the risk of developing extrapulmonary TB (Magee et al., 2016) despite a higher risk of pulmonary TB. Increased hepatic Mtb burden was however observed in the HFD-based murine model of diabetes (Alim et al., 2020), but in this study the animals were infected *via* the intra-venous route and not *via* the natural aerosol route, which likely explain the increased hepatic Mtb burden. HFD-induced alterations in gut microflora leads to severe pulmonary damage and mortality in Toll-like receptor deficient mice (Ji et al., 2014) and it is possible that dysbiosis of the gut microbiota contributes in part to susceptibility of our pre-diabetic animals to TB. In contrast to our observation in HFD-fed mice and those by Alim et al. (2020), HFD-fed guinea pigs with impaired glucose tolerance do not show increased susceptibility to TB and had similar lung Mtb burden to control animals with exception of higher extrapulmonary Mtb burden in the liver 90 days p.i. (Podell et al., 2014). HFD-fed guinea pigs had also similar cytokine profiles in the lung compared to control animals through day 60 p.i., but elevated IL-1β concentrations at 90 days p.i. In the guinea pig model increased susceptibility to TB was only evident in diabetic animals that received a combination of HFD and STZ. These studies highlight distinct species-specific differences in the immune response in animals with dysglycemia.

To determine whether the increased susceptibility of pre-diabetic mice is due to impaired glucose tolerance or obesity, we performed a diet reversal experiment in which we could separate impaired glucose tolerance from obesity. Surprisingly, obese animals with restored glucose tolerance were able to better contain Mtb compared to their healthy-weight controls and had more lung macrophages with a foamy macrophage phenotype. Mtb persists predominantly in a dormant non-replicating state in foamy macrophages compared to infected non-foamy macrophages (Peyron et al., 2008; Russell et al., 2009; Rodriguez et al., 2014). This may explain the overall lower Mtb burden observed in animals with higher foamy macrophage scores. Adiposity and increased foam cell formation has also been suggested to promote latency in humans and contribute to lower TB progression rates in individuals with high BMI (Aibana et al., 2016). The restoration of glucose tolerance, while maintaining a high body fat mass, also resulted in restoration of IFN-γ responses. CCL2 concentrations on the other hand were significantly lower compared to NCD-fed mice. This may serve as a feedback mechanism to limit further recruitment of macrophages to the lung. The change in diet ultimately improved the IFN-γ/IL-10 ratio and necrosis scores were similar in obese animals with a history of glucose impairment compared to healthy chow-fed animals. Observations from our murine model are consistent with findings in humans where obesity in absence of dysglycemia protects against TB (Lonnroth et al., 2010; Lin et al., 2018). A potential limitation of our study is the absence of a control group that continue consumption of HFD for a total of 22 weeks. A published study in mice fed a HFD for 30 weeks showed a small but significant increase (approximately half a log_10_) in lung Mtb burden compared to NCD-fed animals at 14 days p.i., but no differences at later timepoints (Alim et al., 2020). This suggests that the HFD-induced susceptibility modestly increases with duration on HFD and mainly affects early infection with regards to lung Mtb burden.

Taken together, both our HFD-induced pre-diabetes model and the diet reversal model of Mtb infection mimic observations in humans. Our murine models offer the unique opportunity to elucidate the underlying immune-metabolic mechanisms of obesity-induced resistance *vs.* dysglycemia-associated susceptibility to TB. Importantly, our data provide clear evidence, that immune impairment to Mtb including decreased lung IFN-γ production indicative of delayed adaptive immune priming occurs already during pre-diabetes and likely contributes to more severe disease. Future experiments using this model should include investigations of diet-induced changes in immune cell recruitment to the site of infection and cellular immunophenotyping. We further posit that caloric restriction in patients with diabetes or pre-diabetes not only improves glucose tolerance but may also confer at least temporary resistance from TB progression. Thus, large population-based studies are warranted to determine the impact of pre-diabetes and dietary interventions on susceptibility to TB.

## Data Availability Statement

The raw data supporting conclusions of this article will be made available by the authors, without undue reservation.

## Ethics Statement

The animal study was reviewed and approved by The Health Sciences Animal Ethics Committee of The University of Queensland.

## Author Contributions

RS, MDN, and KR wrote the manuscript. RS, MDN, SK, MLD, JK, and AB carried out the experiments. RS, MDN, SB, and HB-O analyzed the data and compiled the figures. RS, MDN, SB, HB-O, SK, SH, AB, CC, KS, and KR interpreted the data and contributed intellectually. All authors contributed to the article and approved the submitted version.

## Funding

This study was supported by grants to KR from the National Institutes of Health (NIH), National Institute of Allergy and Infectious Diseases (NIAID) grant number R01AI116039, the Mater Foundation, the Australian Respiratory Council and the Australian Infectious Diseases Research Centre. KRS received a Fellowship from the Australian Research Council (DE180100512). The Translational Research Institute is supported by a grant from the Australian Government.

## Conflict of Interest

The authors declare that the research was conducted in the absence of any commercial or financial relationships that could be construed as a potential conflict of interest.
