# Supplementary material for: Pre-Diabetes Increases Tuberculosis Disease Severity, While High Body Fat Without Impaired Glucose Tolerance Is Protective
Source: Front Cell Infect Microbiol. 2021 Jul 6;11:691823. doi: 10.3389/fcimb.2021.691823 (PMC8291147; doi:10.3389/fcimb.2021.691823)
Supplement: Supplementary file 1 [file Table_1.docx]

**Supplementary Material**


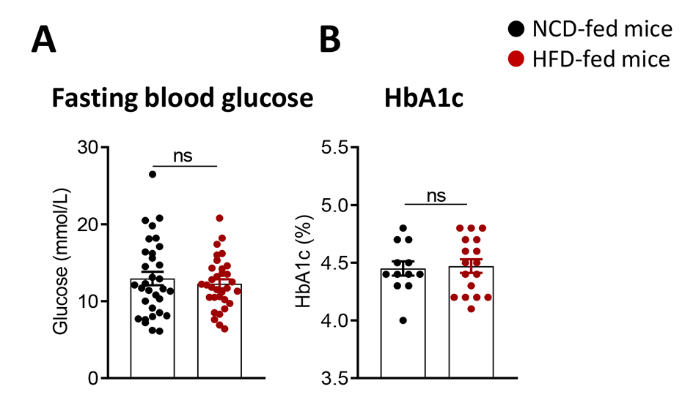


**Figure S1. Characterization of pre-diabetes in C57BL/6 mice.** **A.** Fasting blood glucose was measured after 12 weeks of NCD or HFD (n=30 mice/group analyzed cumulatively across two independent experiments). **B.** Glycated hemoglobin (HbA1c) was measured in whole blood on a randomly selected subset of mice from each group (n=12-17 mice/group analyzed cumulatively across two independent experiments). Data analysis was performed by Mann-Whitney *U* test. ns = not significant.


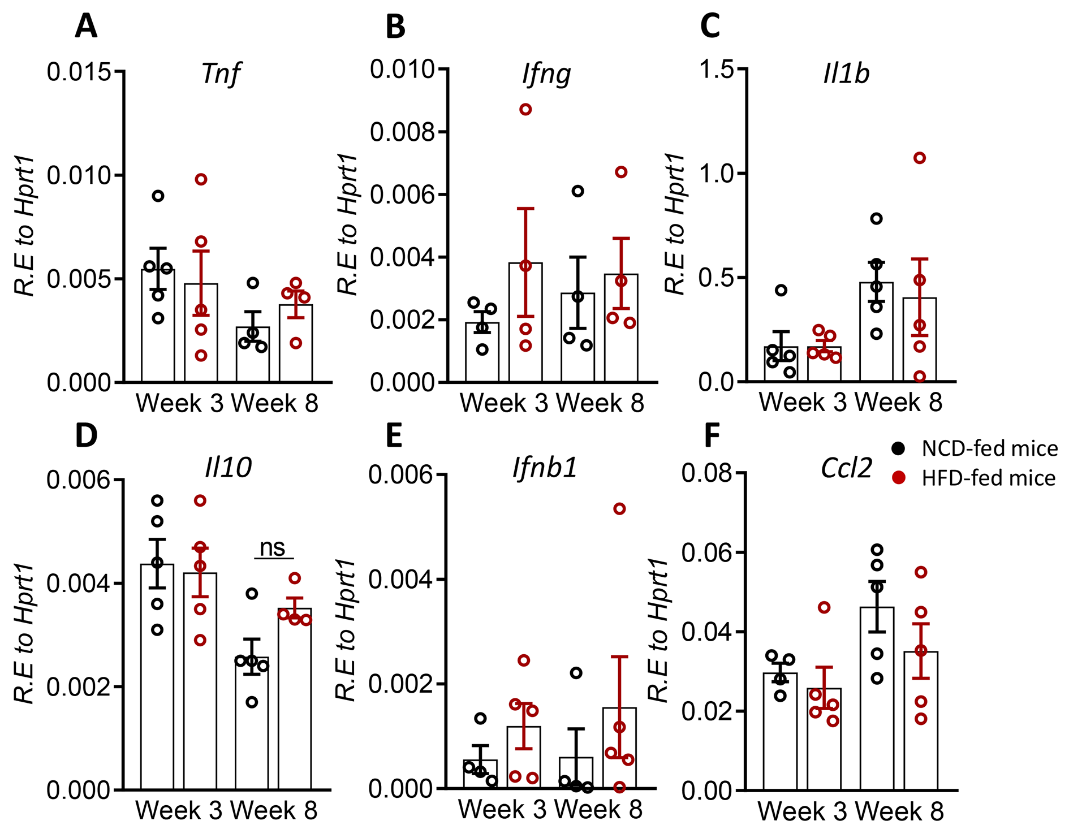


**Figure S2. Inflammatory cytokine mRNA expression in the lung from uninfected NCD- and HFD-fed mice**. Relative expression of **A.** *Tnf*, **B.** *Ifng*, **C.** *Il1b*, **D.** *Il10*, **E.** *Ifnb1* and **F.** *Ccl2*. Data are means ± SEM of n=4-5 mice/group analyzed in one independent experiment. Data analysis was performed by Mann-Whitney *U* test. ns = not significant.


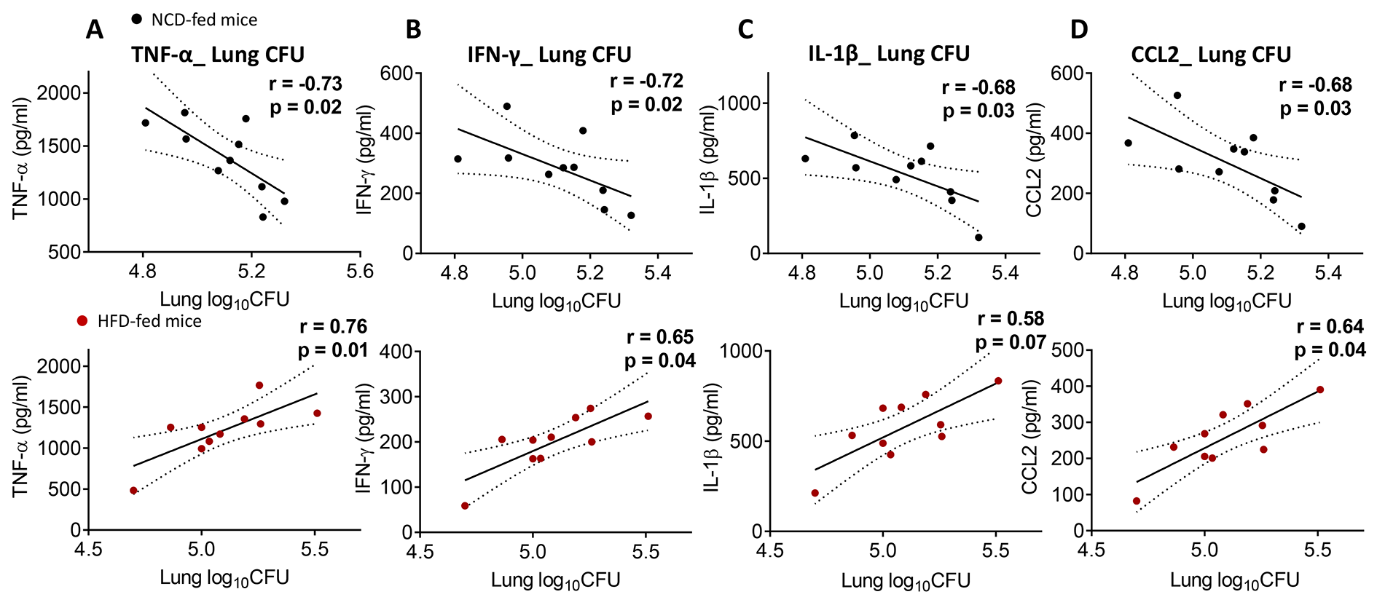


**Figure S3. Correlation between lung bacterial burden and lung pro-inflammatory cytokines/ chemokines in NCD-fed mice and HFD-fed mice. A.** TNF-α, **B.** IFN-γ, **C.** IL-1β and **D.** CCL2 concentrations in NCD or HFD mice correlated with their respective CFUs using the Spearman's rank correlation test. (n=10 mice/group). Spearman r and respective p values have been shown on the figure.


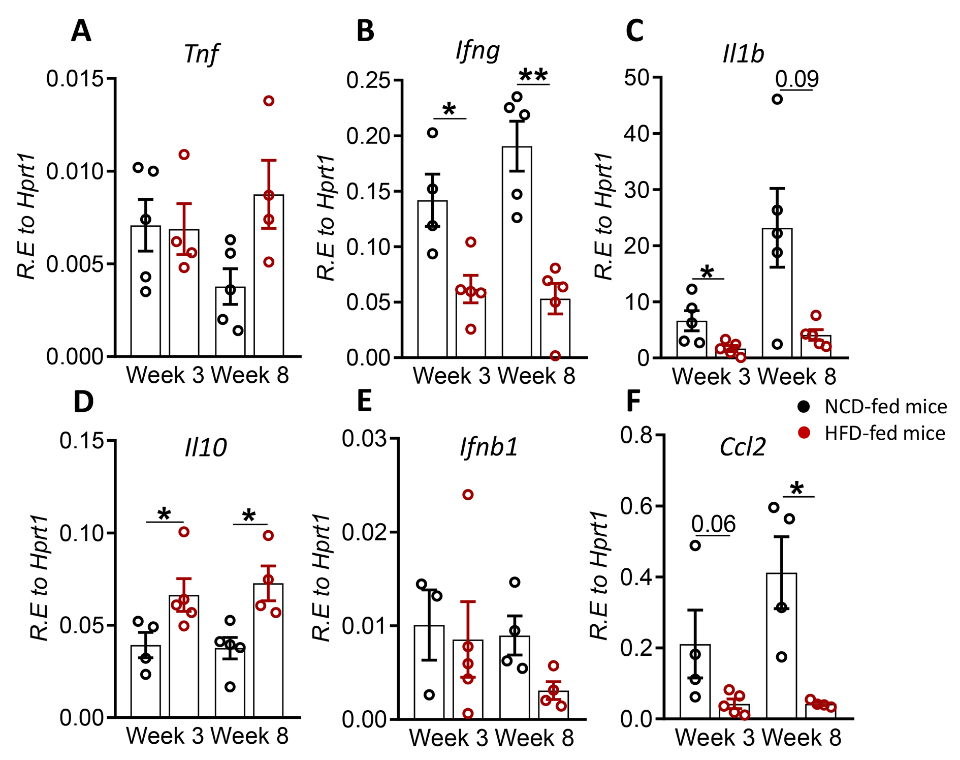


**Figure S4. mRNA expression of inflammatory cytokines in blood from uninfected NCD- and HFD-fed mice**. Relative expression of **A.** *Tnf*, **B.** *Ifng*, **C.** *Il1b*, **D.** *Il10*, **E.** *Ifnb1* and **F.** *Ccl2.* Data are means ± SEM of n=4-5 mice/group analyzed in one independent experiment. Data analysis was performed by Mann-Whitney *U* test. *p < 0.05, **p < 0.01.


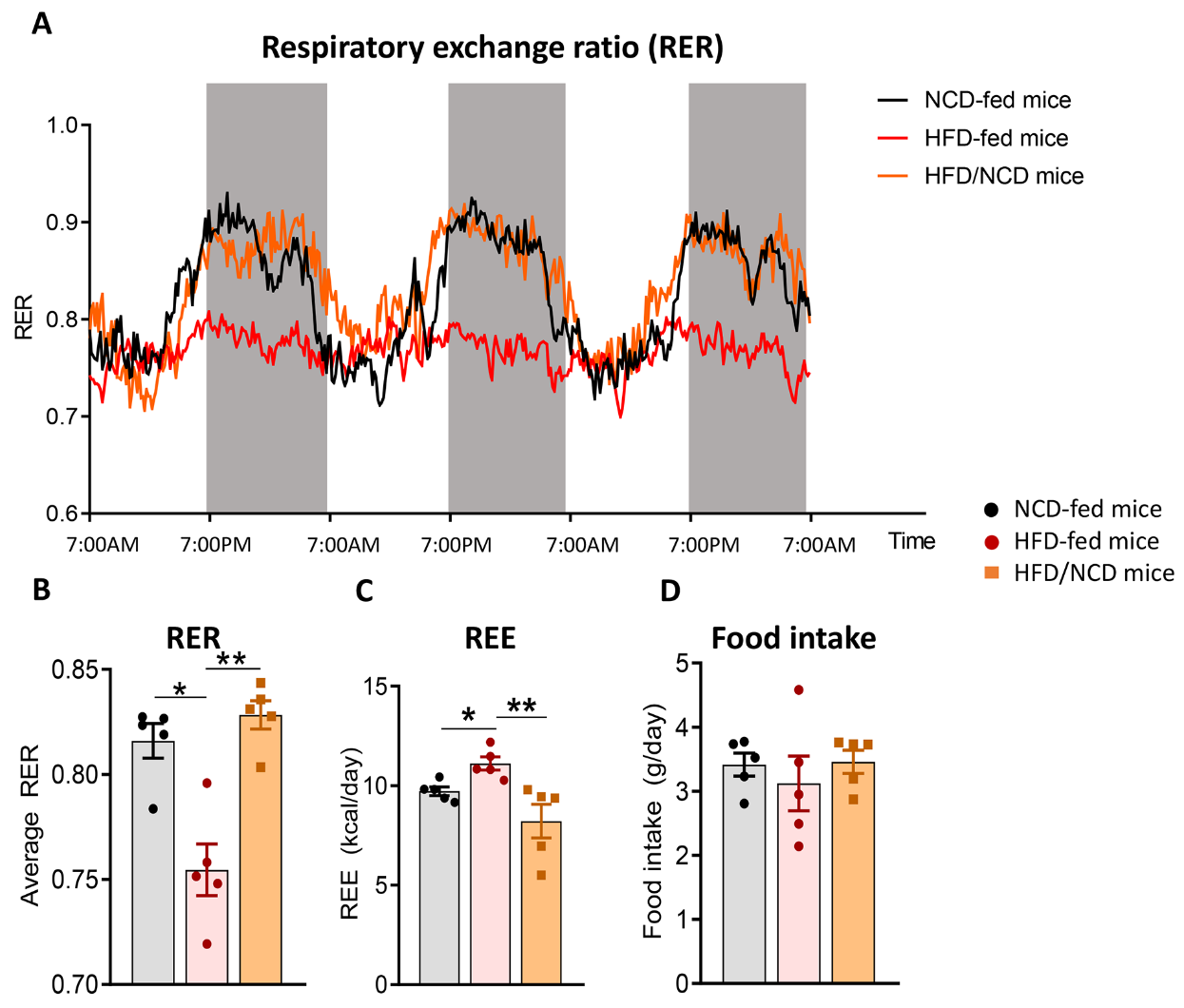


**Figure S5. Metabolic profile of NCD-, HFD- and HFD/NCD mice.** **A.** Changes in RER throughout light/dark phases. Average daily values of **B.** RER, **C.** REE and **D.** food intake. Data are shown as mean ± SEM. (n=5 mice/group). *P < 0.05, **P < 0.01.


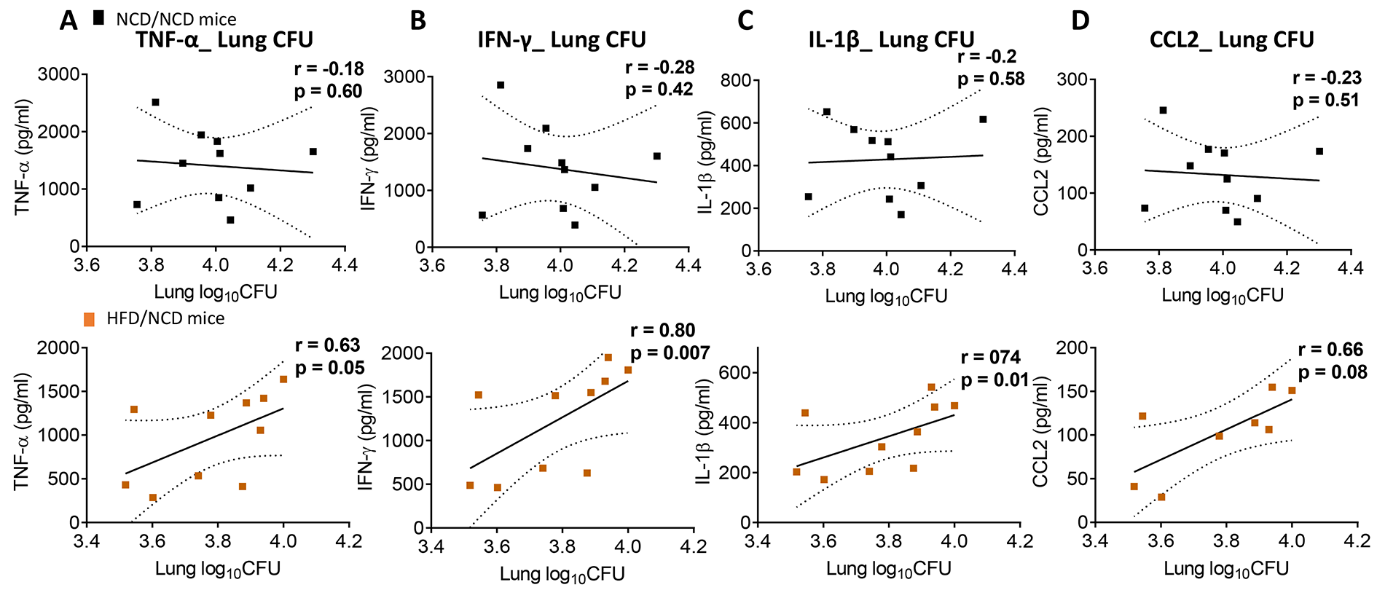


**Figure S6. Correlation between lung bacterial burden and lung pro-inflammatory cytokines/chemokines in NCD/NCD and HFD/NCD mice. A.** TNF-α, **B.** IFN-γ, **C.** IL-1β and **D.** CCL2 concentrations correlated with CFUs using the Spearman's rank correlation test. (n=10 mice/group). Spearman r and respective p values have been shown on the figure.


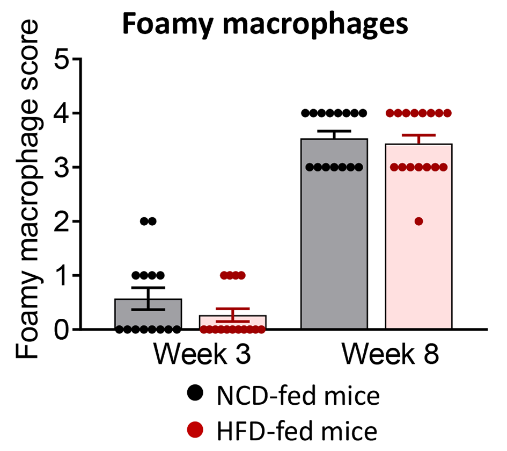


**Figure S7**. **Foamy macrophages in lungs from NCD- and HFD-fed mice.** Histological scoring of appearance of foamy macrophages in lung sections from infected mice at 3 weeks and 8 weeks post-infection. Data represents mean ± SEM (n=10 mice/group analyzed in one independent experiment). Data analysis was performed by Mann-Whitney *U* test.

**Table S1. List of mouse primer sequences**

|  | **Forward (5’-3’)** | **Reverse (5’-3’)** |
| --- | --- | --- |
| *Tnf* | TAGCCCACGTCGTAGCAAAC | ACAAGGTACAACCCATCGGC |
| *Ifng* | CAGCAACAGCAAGGCGAAAAAG | TTTCCGCTTCCTGAGGCTGGAT |
| *Ifnb1* | CGAGCAGAGATCTTCAGGAAC | TCCGCCTCTGATGCTTAAAG |
| *Il1b* | TGGACCTTCCAGGATGAGGACA | GTTCATCTCGGAGCCTGTAGTG |
| *Ccl2* | GCTACAAGAGGATCACCAGCAG | GTCTGGACCCATTCCTTCTTGG |
| *Il10* | CGGGAAGACAATAACTGCACCC | CGGTTAGCAGTATGTTGTCCAGC |
| *Hprt1* | CCCCAAAATGGTTAAGGTTGC | AACAAAGTCTGGCCTGTATCC |
